# Extirpation of the Parotid Gland

**Published:** 1863-08

**Authors:** Daniel Brainard

**Affiliations:** Prof. of Surgery in Rush Medical College


### CHICAGO MEDICAL JOURNAL.

VOL. VI.] AUGUST, 1863. [No. 8.

### ORIGINAL COMMUNICATIONS.

### EXTIRPATION OF THE PAROTID GLAND.

By DANIEL BRAINARD, M. D., Prof. of Surgery in Rush Medical College.

Timothy Bradley, of Fond du Lac, Wisconsin, aged 45, healthy, of good constitution, perceived when he was 21 years of age a tumour below the body of the lower jaw. This grew to the size of his "fist" without pain, and was removed in 1850 in Ireland.

About 1858 he perceived it returning in a small tumour behind the ramus of the jaw on the right side. It grew without pain until Jan. 1863, when it presented the appearance shown in the photographic figure. It then extended up to the zygomatic arch, and down to the middle of the neck, forward upon the side of the face, and backwards under the sterno-mastoid muscle, was detached, very moveable, but the skin was adherent to the surface.

Wednesday, Jan. 14th, 1863, I removed it in presence of the Medical Class of Rush Medical College, assisted by Prof. J. W. Freer.

Two incisions were made to embrace the adherent portion of the skin, which was then dissected up before and behind. I then commenced separating it from below upwards with the finger. This was readily done till the back and upper part was reached where it involved the external carotid and jugular vein, which were tied below and then divided. The dissection was then completed mostly with a blunt instrument. The upper end of the external carotid artery required ligature, and one branch below. The tumor in its growth had drawn the parotid gland out of its place so that it was not difficult to pass an instrument behind its upper part.

When the tumor was removed, there was a space extending from the articulation of the lower jaw below the corner of the Os-Hyoides. The styloid process, stylo-hyoid ligament, the internal jugular vein and internal cerebral artery were exposed, and the space behind and within the ramus of the jaw was cleared.

Pro. Freer, for many years Prof. of Anatomy in the College, examined carefully and could find no trace of the Parotid gland. The right side of the face was paralyzed.

On examination of the tumor, pieces of the gland in a healthy state were found around the upper edge, below this a considerable part seemed composed of the same tissue altered in structure which was softened and redder than natural. At the lower part there was a softer granulated mass, which Dr. Freer examined with the microscope. He found no common cells, but rounded granules with traces of ducts.

Without assuming to decide positively as to the tissue in which this disease originated, it is certain that it involved the whole of the Parotid gland except slight particles above.

To the naked eye the structure of it appeared to be the fibro splastic material. No doubt can, I think, exist as to the removal of the entire gland, which I have removed in two other instances, and the reports of which cases have been heretofore published in this Journal.

The time required to complete the operation was perhaps thirty minutes. The hæmorrhage was considerable, but by tying the external carotid before dividing it, this was partly controlled. No accident happened to the patient, and in twenty days he returned home with the wound nearly healed.

